# Epigenetic Modifications in Placenta are Associated with the Child's Sensitization to Allergens

**DOI:** 10.1155/2019/1315257

**Published:** 2019-04-17

**Authors:** Hani Harb, Bilal Alashkar Alhamwe, Nathalie Acevedo, Paolo Frumento, Catharina Johansson, Lisa Eick, Nikos Papadogiannakis, Johan Alm, Harald Renz, Daniel P. Potaczek, Annika Scheynius

**Affiliations:** ^1^Institute of Laboratory Medicine, Member of the German Center for Lung Research (DZL) and the Universities of Giessen and Marburg Lung Center (UGMLC), Philipps-University Marburg, Germany; ^2^International Inflammation (in-VIVO) Network, Worldwide Universities Network (WUN), West New York, NJ, USA; ^3^Division of Immunology, Boston Children's Hospital, Harvard Medical School, Boston, MA, USA; ^4^College of Pharmacy, International University for Science and Technology (IUST), Daraa, Syria; ^5^Department of Clinical Science and Education, Karolinska Institutet, and Sachs' Children and Youth Hospital, Södersjukhuset, Stockholm, Sweden; ^6^Institute for Immunological Research, University of Cartagena, Cartagena, Colombia; ^7^Unit of Biostatistics, Institute of Environmental Medicine, Karolinska Institutet, Stockholm, Sweden; ^8^Department of Laboratory Medicine, Division of Pathology, Section of Perinatal Pathology, Karolinska Institutet and Karolinska University Hospital Huddinge, Stockholm, Sweden; ^9^John Paul II Hospital, Krakow, Poland; ^10^Clinical Genomics, Science for Life Laboratory, Stockholm, Sweden

## Abstract

Prenatal environmental exposures are considered to contribute to the development of allergic sensitization by epigenetic mechanisms. The role of histone acetylation in the placenta has not been examined yet. We hypothesized that placental histone acetylation at the promoter regions of allergy-related immune regulatory genes is associated with the development of sensitization to allergens in the child. Histones H3 and H4 acetylation at the promoter regions of 6 selected allergy-related immune regulatory genes was assessed by a chromatin immunoprecipitation assay in 173 term placentas collected in the prospective birth-cohort ALADDIN. The development of IgE sensitization to allergens in the children was followed from 6 months up to 5 years of age. We discovered significant associations of histone acetylation levels with decreased risk of allergic sensitization in 3 genes. Decreased risk of sensitization to food allergens was associated with higher H3 acetylation levels in placentas at the* IFNG* and* SH2B3 *genes, and for H4 acetylation in* HDAC4*. Higher* HDAC4 *H4 acetylation levels were also associated with a decreased risk of sensitization to aeroallergens. In conclusion, our results suggest that acetylation of histones in placenta has a potential to predict the development of sensitization to allergens in children.

## 1. Introduction

During the last decades the prevalence of allergic sensitization and allergic diseases has increased worldwide, particularly in children [1, 2]. One hypothesis to explain this allergy epidemic is that environmental and lifestyle conditions modify the epigenome of immune cells, so the immune response is skewed to proallergic profiles [3–5]. Prenatal or early childhood exposures to environmental factors, such as living in urban or rural/farming areas can affect the programming of the immune system and are thus risk factors for development of subsequent allergic diseases [6]. Epigenetic modifications have been postulated as an important mechanism mediating these effects [7–10], which has been demonstrated mostly by human studies investigating DNA methylation [11, 12]. Previously, it has been found that DNA methylation levels within the* CD14* promoter region are lower in placentas of mothers living on a farm, suggesting that epigenetic regulation of* CD14* early in life might be involved in the protective effect of “living on a farm”, with regard to allergy development [13]. 

Much less in this context is known on the eventual role of epigenetic modifications of histones in allergy development. Unlike DNA methylation, histone modifications, for example, histone acetylation, methylation, or phosphorylation, are biochemical changes affecting not the nucleic acid itself but lysine residues on histones. Increased acetylation of histones H3 and H4 is typically associated with better accessibility of promoters to transcriptional machinery and thereby higher gene expression [5]. Previous studies have shown that changes in histone acetylation levels can affect polarization of T helper type 2 (Th2) cells/response [14].

In the present study, we hypothesized that acetylation of H3 or H4 histones in the promoter regions of potentially allergy-related immune regulatory genes in placenta tissue is associated with the development of sensitization to food and airborne allergens in the child early in life. We nested our study within the prospective birth-cohort ALADDIN (Assessment of Lifestyle and Allergic Disease During INfancy), which consists of families with different lifestyles, anthroposophic, partly anthroposophic or conventional [15]. Anthroposophic lifestyle is mainly characterized by organic diet with live lactobacilli, restrictive use of antibiotics and vaccine, and home delivery [15, 16]. The ALADDIN cohort was designed to elucidate why children in anthroposophic families are less sensitized to allergens compared to those living in families with a conventional lifestyle [15, 16]. The results might indicate that acetylation of histones in allergy-related immune regulatory genes in placenta has a potential to predict the development of sensitization to allergens in children early in life.

## 2. Materials and Methods

### 2.1. Study Population: The ALADDIN Cohort

Mothers and their children in this study are part of the prospective birth-cohort ALADDIN, which consists of families with different lifestyles [15, 16]. A total of 330 families were recruited at anthroposophic and conventional healthcare centers in the Stockholm area between September 2004 and November 2007. Families were enrolled in the study at gestational weeks 25-37 (median 30). The lifestyle groups were classified based on choice of maternal-child health centers and parental responses to a questionnaire two months after the birth of the child as described in more detail elsewhere [15]. Inclusion criteria for the present study were not severe illness before or during pregnancy, ≥36 weeks of gestation, and availability of snap frozen placenta specimens stored at -80°C which had not previously been thawed for use in other studies ending with 173 placentas (Table 1). This study was conducted in accordance with the Declaration of Helsinki and was approved by the Regional Ethical Review Board in Stockholm (project Dnr 2010/1811-32). All parents gave their written informed consent for inclusion before they participated in the study.

### 2.2. Determination of Allergen Sensitization

Blood samples were obtained from parents at inclusion in the study and from the child at 6, 12, 24, and 60 months of age. Samples were collected in heparin tubes and plasma was stored at -20°C. Parental sensitization was analyzed by ImmunoCAP Phadiatop™ for IgE to a mix of 11 aeroallergens. Available blood samples from the children at 6, 12, and 24 months of age were analyzed by ImmunoCAP tests for IgE to cow's milk, hen's egg, peanut, cat, dog, birch, and timothy. At 60 months, a food mix (fx5) and Phadiatop™ were used. If fx5 was positive, the allergens cow's milk, hen's egg, peanut, codfish, wheat flour, and soybean were separately analyzed and if Phadiatop™ was positive, cat, dog, horse, birch, timothy, mugwort,* Cladosporium*,* Dermatophagoides farinae*, and* D. pteronyssinus* (all kits from Thermo Fisher Scientific, Uppsala, Sweden). Allergen specific IgE levels ≥0.35 kU_A_/L were categorized as IgE sensitization.

### 2.3. Collection of Placenta Specimens and Histopathologic Examination

The placentas (n=173) were collected by midwifes directly after birth, put on ice, and sent to the Karolinska University Hospital Solna. From each placenta, a cross-sectional sample about 0.5 cm thick, 1.5 cm wide, and spanning the whole thickness of the placenta was cut near the umbilical cord, quickly washed two times in phosphate buffered saline (PBS) to remove as much blood as possible and then snap frozen on dry ice and stored at -80°C. These samples were later used for histone acetylation analyses.

The placentas were also subjected to routine histopathological examination. Two placental tissue biopsies, one from the vicinity of the umbilical cord and one from the periphery of the placenta, about 0.5 cm thick and spanning the whole thickness of the placenta, a piece of the membranes, and a piece of the umbilical cord were obtained. All specimens were washed with PBS, fixed in formalin, paraffin-embedded, and evaluated on routine haematoxylin and eosin-stained sections. In two cases inadequate material had been sampled leaving 171 placentas to be finally analyzed for histopathology. An experienced perinatal pathologist (NP) who was blinded to the demographic data for the participating families (see Table 1) examined all slides. The presence of chorioamnionitis, vasculitis, funisitis, and villitis was recorded. Chorioamnionitis, irrespective of grading, was defined as presence of polymorphonuclear leucocytes in subchorionic plate or in amniochorion. Vasculitis was defined as the presence of leukocytes in the vessel wall of chorionic plate or umbilical vessels. Funisitis was defined as the presence of leukocytes in Wharton's jelly. Villitis was defined as the presence of mononuclear cell infiltrates in the villous stroma [17].

### 2.4. Selection of Genes for Acetylation Analyses in Placenta

*CD14 *(CD14 molecule),* FOXP3 *(Forkhead box P3)*, HDAC4* (Histone deacetylase 4),* INFG *(Interferon gamma), and* IL13* (Interleukin 13) were selected based on (1) previous data showing significant differences in DNA methylation between atopic and nonatopic children [18], (2) empirical evidence of differential DNA methylation due to exposure to environmental factors such as farming [13], (3) other studies showing potential allergy-relevant association on the epigenetic level [19, 20], and (4) evidence in various allergy-related animal models of epigenetic changes that could be transmitted to offspring [21, 22].* SH2B3* (SH2B adaptor protein 3) was selected based on the results of a genome-wide DNA methylation study revealing significant differences in the DNA methylation levels of this gene in purified memory cutaneous lymphocyte-associated antigen (CLA)^+^ T cells from atopic eczema patients [23].

### 2.5. Isolation of Chromatin from Snap Frozen Placenta, Chromatin Immunoprecipitation, and Quantitative Polymerase Chain Reaction

A subsection spanning the whole thickness of the placenta was manually obtained in a -80°C freezer from the original sample and then kept for 8 min in 1 ml 1% paraformaldehyde (PFA; Sigma-Aldrich, Munich, Germany) at room temperature (RT). Next, the sample was centrifuged for 5 min at 7,870* g* at RT, incubated with 1 ml 0.25% trypsin-ethylenediaminetetraacetic acid (EDTA; Thermo Fisher Scientific, Waltham, MA, USA) for 1 hour at RT and then again centrifuged for 5 min at 7,870* g* at RT. The supernatant was discarded, and the tissue components were incubated with 0.1% collagenase (Roche Diagnostics, Mannheim, Germany) for another hour at RT and then centrifuged again for 5 min at 7,870* g* at RT. To purify cells from tissue remnants and cell debris, the pellet was then resuspended in 1 ml PBS and run through a 0.2 *μ*m sieve. Next, the cells were washed twice with 1 ml PBS. Further steps, including chromatin purification, chromatin immunoprecipitation (ChIP), and quantitative assessment of H3 or H4 histone acetylation by polymerase chain reaction (PCR), were conducted as established and thoroughly validated before [24]. PCR primers used in the present study are given in (Table 2).

In brief, three-level strategy of PCR data normalization was applied. First, percent enrichment to the input control was calculated for each target locus and a positive control gene encoding ribosomal protein L32 (*RPL32*), separately for mock (IgG), H3, and H4 antibodies. Then, locus-specific percent enrichment to the input control obtained for IgG was subtracted from the corresponding values for H3 or H4 antibodies. Such calculated IgG-corrected percent enrichment was divided for each gene into that of* RPL32* resulting in a relative enrichment value, which was used for subsequent statistical analyses [24, 25]. Intra- and interassay coefficients of variation calculated for percent enrichment should not exceed 10% [24]. All samples were processed according to the same standardized protocol and analyzed blinded and in a randomized order.

### 2.6. Statistical Analyses

Due to the limited number of available placenta specimens in the anthroposophic group, this group was merged with the partly anthroposophic group for the statistical analyses. Demographic data were compared between the study subgroups either by Fisher's exact test (binary variables) or Mann-Whitney-Wilcoxon rank sum test (continuous variables). Fisher's exact test was used in the analyses of placenta histopathology in relation to lifestyle and sensitization of the children and their sex. The histone acetylation levels were presented by their median and interquartile range in the different lifestyle groups, and Mann-Whitney-Wilcoxon rank sum test was used to compare groups.

Binary variables indicating sensitization to food- or aeroallergens were recorded at 6 months, 1, 2, and 5 years of age. Generalized estimating equations (GEE) were used to compute odds ratios (ORs) associated with histone acetylation levels and the corresponding 95% CIs. All regressions included dummy variables indicating the time, in order to capture potential nonlinear trends. Additional analyses were performed by further adjusting for the sensitization of the parents. OR reflects the change in the odds of being sensitized associated with a unit increase of the acetylation levels. An OR greater than 1 indicates that the associated predictor may be a risk factor for sensitization; an OR less than 1 suggests that the associated predictor is protective against sensitization; an OR equal to 1, or not significantly different from it, does not permit establishing an association between histone acetylation levels and the risk of sensitization. The R package gee, version 4.13-19 (https://cran.r-project.org/web/packages/gee/), was used for the analysis. The analyses were repeated stratifying by gender and, separately, by lifestyle. A p value < 0.010 was considered significant. Model-based receiver operating characteristic (ROC) curves were drawn to test for the ability to predict sensitization to allergens and the area under the curve (AUC) was calculated as a measure of performance, using the GEE logit model described above.

## 3. Results

### 3.1. Study Population

Comparison of the lifestyle groups showed significant differences for the anthroposophic (anthroposophic + partly anthroposophic) lifestyle characteristics compared with the nonanthroposophic group regarding a lower prevalence of sensitization, particularly to food allergens, in the children (Table 1).

### 3.2. Placenta Histopathology

Placental histopathology showed neither significant differences between the two-lifestyle groups (Table 3(a)) nor any associations with sensitization to allergens in the children between 6 months up to 5 years of age (Table 3(b)), irrespective of the child's sex (Supplementary Tables 1A and 1B).

### 3.3. Associations between Demographic Data and Placental Histone Acetylation

The age of the mother, parity or parental sensitization to aeroallergens showed no significant associations with histone acetylation levels in the placentas, nor did the child's sex, birth weight, or the gestational age at delivery (data not shown). In addition, there was no significant effect of the lifestyle on the placental histone acetylation levels and also none when prestratified for the child's sex (Supplementary Table 2).

### 3.4. Placental Histone Acetylation and Reduced Risk of Allergic Sensitization in the Child

**Figure 1:**
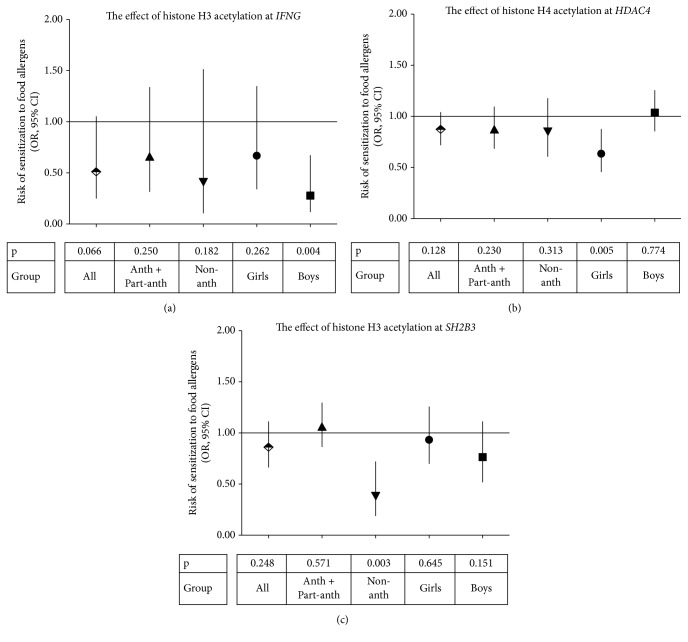
The association between placental tissue histone acetylation levels at promoter regions of (a)* IFNG* (H3), (b)* HDAC4* (H4), and (c)* SH2B3* (H3) genes and the risk of sensitization to food allergens in children. For the methodology of statistical calculations, please, see Methods. Anth + Part-anth denotes a combined anthroposophic and partly anthroposophic lifestyle group and Non-anth the nonanthroposophic lifestyle. OR denotes odds ratio; 95% CI, confidence interval.

**Figure 2:**
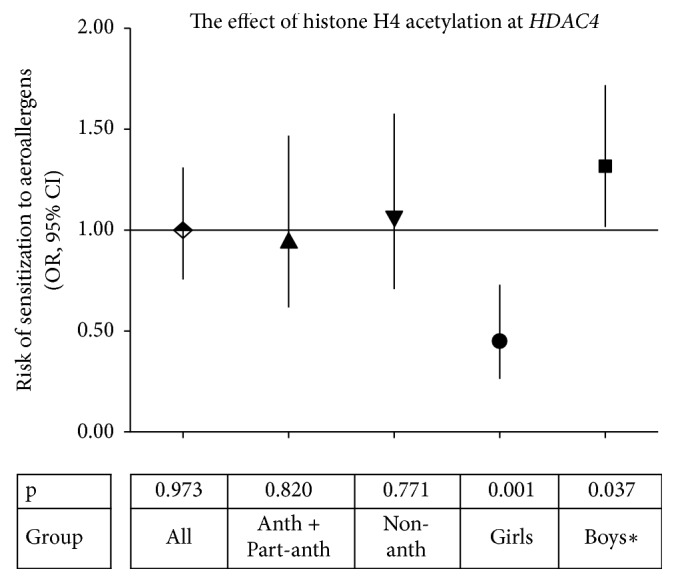
The association between placental tissue histone H4 acetylation levels at promoter region of the* HDAC4* gene and the risk of sensitization to aeroallergens in children. For the methodology of statistical calculations, please, see Methods. For abbreviations, see Figure 1. *∗*Calculated with logistic regression since the number of observations in the group of boys precluded the estimations of OR and CI with the GEE model.

Placental histone acetylation levels at the promoter regions of 3 genes,* IFNG, HDAC4, *and* SH2B3, *turned out to be predictive for the development of allergic sensitization in the children followed longitudinally from 6 months up to 5 years of age. Decreased risk of sensitization to food allergens was associated with higher H3 acetylation levels in placentas at the* IFNG* loci in male offspring and to a higher H4 acetylation at the* HDAC4* promoter in female offspring (Figures 1(a) and 1(b)). In addition, higher H3 acetylation at the* SH2B3* locus was associated with a decreased risk of sensitization to food allergens in children born in nonanthroposophic families (Figure 1(c)). Regarding sensitization to aeroallergens, a higher H4 acetylation level at the* HDAC4* promoter decreased the risk of sensitization in female offspring (Figure 2). All these associations remained significant after adjustment to either maternal or paternal allergic sensitization. We did not observe any significant associations between histone acetylation levels at the* CD14*,* IL13,* and* FOXP3* promoters and the development of sensitization to allergens (data not shown).

**Figure 3:**
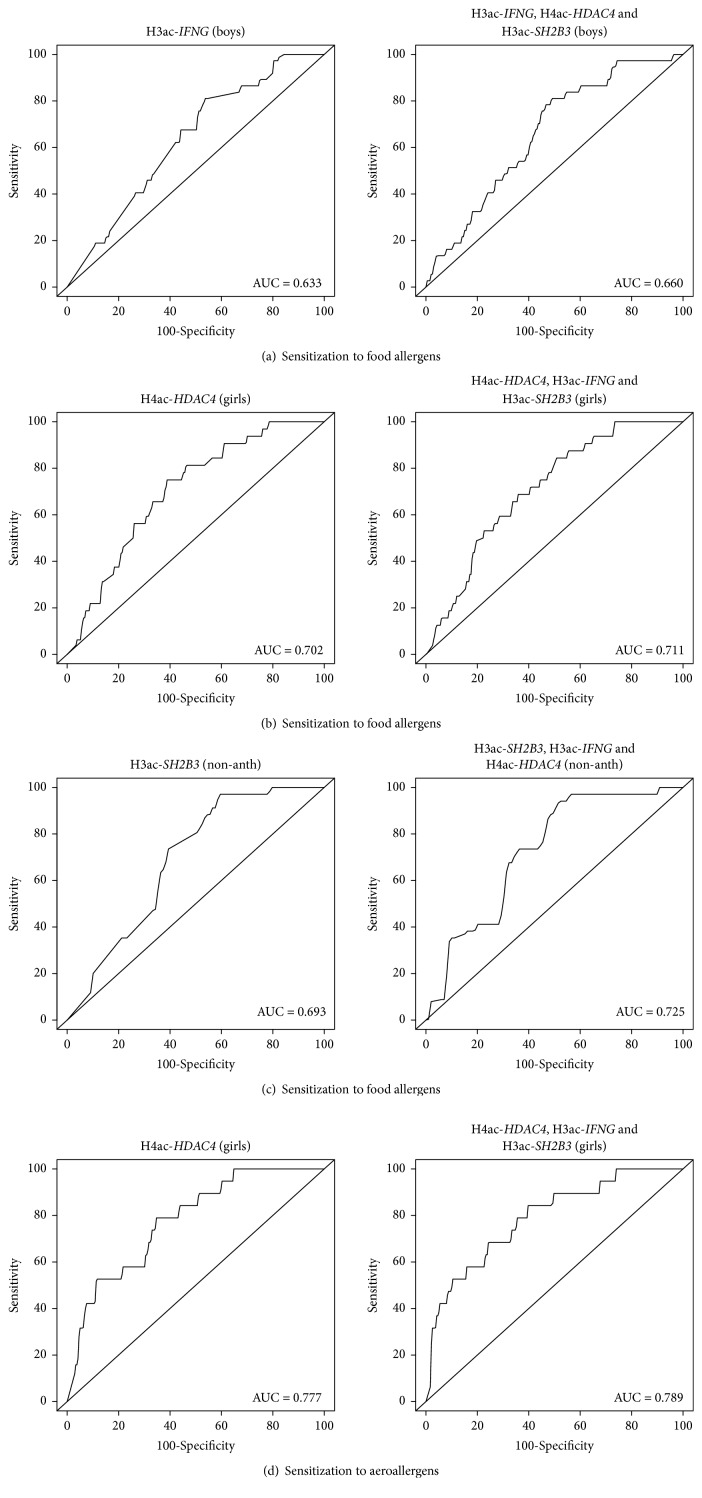
Receiver operating characteristic (ROC) curves and area under the curve (AUC) obtained from different logistic regression models implemented through GEE to predict the development of sensitization to allergens.* Left panel*: (a) H3 acetylation (H3ac) at the* IFNG* promoter versus sensitization to food allergens in boys, (b) H4 acetylation (H4ac) at the* HDAC4* promoter in girls versus sensitization to food allergens in girls, (c) H3 acetylation (H3ac) at the* SH2B3* locus versus sensitization to food allergens in nonanthroposophic children (non-anth), and (d) H4 acetylation (H4ac) at the* HDAC4* promoter versus sensitization to aeroallergens in girls.* Right panel*: (a-d), analogous prediction obtained when the GEE logistic model is further adjusted for the other histone acetylation variables showing significant associations with sensitization to allergens, as indicated (see also Figures 1 and 2).

Next, we computed model-based ROC curves to see how well the used regression models can predict sensitization to allergens and computed AUC as a measure of performance. On the left panels in Figure 3, we report the following ROC curves: H3 acetylation at the* IFNG* promoter versus sensitization to food allergens in boys (Figure 3(a)), H4 acetylation at the* HDAC4* promoter in girls versus sensitization to food allergens in girls (Figure 3(b)), H3 acetylation at the* SH2B3* locus versus sensitization to food allergens in nonanthroposophic children (Figure 3(c)), and H4 acetylation at the* HDAC4* promoter versus sensitization to aeroallergens in girls (Figure 3(d)). The ROC curves and the AUC suggest that the predictive power of the models is rather limited with the highest AUC level of 0.777 (Figure 3(d)). In the right panels in Figure 3, the ROC curves obtained with the GEE logistic model are further adjusted for the other histone acetylation variables showing any significant associations with sensitization to allergens (see Figures 1or 2, respectively). Results show that multiple adjustment does not significantly improve the predictive power.

The dataset for this study including demographic data, sensitization to allergens, and histone acetylation levels can be found in the* Supplementary data table*.

## 4. Discussion

This is the first study linking the development of sensitization to allergens early in life with defined changes in the histone acetylation of important immunoregulatory genes in the placenta. We have discovered significant associations between histone acetylation levels in 3 of the 6 allergy candidate genes examined in placentas with the development of sensitization to allergens.

Higher placental histone acetylation levels were associated with decreased risk of allergic sensitization to food allergens in children. This involved H3 acetylation in the* IFNG* gene, H4 acetylation in* HDAC4*, and H3 acetylation in* SH2B3*. Furthermore, H4 acetylation in* HDAC4 *was also associated with a decreased risk of allergic sensitization to aeroallergens. These findings revealed* HDAC4* and* SH2B3* as two new candidates implicated in the susceptibility to allergic sensitization. The protein encoded by* HDAC4 *possesses histone deacetylase activity and represses transcription when tethered to a promoter [26, 27]. This protein does not bind to DNA directly, but through transcription factors MEF2C and MEF2D and it seems to interact in a multiprotein complex with HDAC3 and RB binding protein 4 (RBBP4), a molecule which may target histone deacetylases to their histone substrates [26]. HDAC4 is of great interest in B cell biology since forced expression of HDAC4 impairs the inflammatory effects of miRNA-155 in this cell [28] and because B cell functions seems to be particularly sensitive to HDAC inhibitors [29]. HDAC4 also provides deacetylase activity for nonhistone proteins in the cytoplasm including signal transducer and activator of transcription 1 (STAT1), a protein that promotes interferon signaling pathways [30]. We speculate that increased H4 acetylation of* HDAC4* detected in this study may lead to changes in* HDAC4* expression in placenta and by genome-wide deacetylase effects alter the expression of other immune genes and/or transcription factors involved in Th1-skewing which in turns facilitates early immune polarization and could confer protection from IgE sensitization in the offspring.* HDAC4* is an age-modified locus [26] and has shown to be susceptible to epigenetic modification by environmental exposures including supplementation with n-3 PUFAs [31].

*SH2B3* encodes a member of the SH2B adaptor family of proteins that acts as a key negative regulator of cytokine signaling. It plays a critical role in lymphohematopoiesis, inflammation [32], and IL7R signaling in B cell progenitors [33]. It also regulates granulocyte-macrophage colony-stimulating factor (GM-CSF) and interleukin 15 (IL-15) signals in mature dendritic cells (DCs) and affects their ability to prime naive CD4^+^ T cells towards IFNG production [34]. Our results support significant association of acetylation differences in the* IFNG* gene in placenta and the decreased risk of allergic sensitization, particularly to food allergens (Figure 1(a)). Immune-homeostasis after birth depends on a rapid development of strong T helper type 1 (Th1) responses. These are needed to combat extracellular and intracellular pathogens and help to keep the development of pathogenic Th2 immune responses down. It has previously been shown [33, 35, 36] that children protected from the development of (respiratory) allergy have already, at birth, high levels/production of Th1 related cytokines, including IFNG. We are now able to extend these observations and show for the first time that already during pregnancy such a response seems to develop at least at the late stage of gestation in the placental tissue.

Several of our observations were gender-specific; for instance, H4 acetylation differences were only significant for girls, in agreement with accumulating evidence on gender-specific effects of environmental exposures in placenta. Sex-specific epigenetic effects have been also reported in the context of allergies by others, but a functional and mechanistic explanation for these findings is still missing [37, 38].

A limitation in this study is that only 25 anthroposophic families could be included due to the amount of available snap frozen placenta specimens in this life style group (Table 1). We therefore fused the anthroposophic group with the partly anthroposophic group for the statistical analyses that is why separate life style analyses was hampered. Another limitation is the use of placenta tissue, where the cell heterogeneity in the samples does not allow any interpretation to which particular cells the epigenetic profiles of H4 and H3 acetylation could be ascribed. We performed, however, careful placenta histopathology examinations to address any bias due to inflammation and could exclude any significant differences in the presence and distribution of leukocytes between the lifestyle groups or any associations to sensitization to allergens in the children (see Tables 3(a) and 3(b)). Furthermore, since we analyzed global H3 and H4 acetylation levels in each of the genes it is not possible to discriminate specific marks, which underlie the associations detected in this study. The combinations of genes and histone acetylation marks analyzed in this study demonstrated rather moderate predictive value for the development of sensitization to allergens. Deeper and fine-tuned mapping of histone marks as well as whole genome sequencing is needed to delineate the exact predictive effect of different genes.

## 5. Conclusions

Our results indicate that histone acetylation levels in allergy-related immune regulatory genes in placenta might have a potential to predict the development of sensitization to allergens in the child within the first 5 years after birth. The epigenetic profiles shown here for the acetylation in the H3 and H4 histones may open new preventive avenues and lay the foundation for further prospective studies.

## Figures and Tables

**Table 1:** Demographic data for the participating families and sensitization to allergens.

	Anthroposophic N=25	Partly anthroposophic N=105	Anthroposophic and partly anthroposophic N=130	Non-anthroposophic N=43	p^*∗*^
*Parents*					

Mother's age (years)	30 (23-32)	31 (28-34)	31 (27-34)	30 (28-33)	0.468
Mother sensitized to aeroallergens^a^	5/25 (20%)	29/105 (28%)	34/130 (26%)	14/43 (33%)	0.537
Father sensitized to aeroallergens^a^	8/23 (35%)	45/100 (45%)	53/123 (43%)	14/40 (35%)	0.473

*Mother during pregnancy*					

Parity					
First	7/25 (28%)	46/103 (45%)	53/128 (41%)	17/43 (40%)	0.971
Second	9/25 (36%)	40/103 (39%)	49/128 (38%)	18/43 (42%)	0.814
Third (fourth or fifth)	9/25 (36%)	17/103 (17%)	26/128 (20%)	8/43 (19%)	0.982

*Child*					

Sex (female)	13/25 (52%)	52/105 (50%)	65/130 (50%)	28/43 (65%)	0.122
Birth weight (g)	3550 (3355-3760)	3585 (3345-3950)	3568 (3348-3939)	3510 (3312-4010)	0.648
Gestational age at birth (completed weeks)	40 (39-41)	40 (39-41)	40 (39-41)	39 (38-40)	0.009

*Child sensitized to*					

Food allergens at (Girls n/N; Boys n/N % p*∗*)					
6 mo^b^ (4/70 5.7; 6/63 9.5 p=0.52)	0/16 (0%)	4/84 (5%)	4/100 (4%)	6/33 (18%)	0.022
12 mo^b^ (8/68 11.8; 10/59 16.9 p=0.45)	0/13 (0%)	9/77 (12%)	9/90 (10%)	9/37 (24%)	0.068
2 yrs^b^ (7/68 10.3; 13/62 21.0 p=0.14)	2/15 (13%)	9/79 (11%)	11/94 (12%)	9/36 (25%)	0.108
5 yrs^c^ (13/60 21.7; 8/52 15.4 p=0.47)	1/16 (6%)	10/69 (14%)	11/85 (13%)	10/27 (37%)	0.012
Aeroallergens at					
6 mo^d^ (3/64 4.7; 0/53 0.0 p=0.25)	0/13 (0%)	2/72 (3%)	2/85 (2%)	1/32 (3%)	0.999
12 mo^d^ (2/65 3.1; 2/56 3.6 p=1.0)	0/12 (0%)	1/75 (1%)	1/87 (1%)	3/34 (9%)	0.120
2 yrs^d^ (3/68 4.4; 6/60 10.0 p=0.30)	0/15 (0%)	4/77 (5%)	4/92 (4%)	5/36 (14%)	0.130
5 yrs^e^ (11/61 18.0; 11/53 20.8 p=0.81)	1/16 (6%)	13/71 (18%)	14/87 (16%)	8/27 (30%)	0.201

Categorical variables: n/N yes/total number (%). Continuous variables: median (interquartile range).

*∗*p for comparisons of combined anthroposophic and partly anthroposophic versus nonanthroposophic lifestyle group and for girls *vs *boys regarding sensitization to allergens. Categorical variables: Fisher's exact test; continuous variables: Mann-Whitney-Wilcoxon rank-sum test.

^a^Classified as sensitized if IgE level was ≥0.35 kU_A_/L measured using Phadiatop™ (Thermo Fisher Scientific) a mix of 11 aeroallergens.

^b^Classified as sensitized if IgE level was ≥0.35 kU_A_/L for at least one of the three food allergens analyzed using ImmunoCAP™ (Thermo Fisher Scientific).

^c^Classified as sensitized if IgE level was ≥0.35 kU_A_/L for at least one of the six food allergens analyzed using a food mix, fx5, followed by separate ImmunoCAP™ tests (Thermo Fisher Scientific).

^d^Classified as sensitized if IgE level was ≥0.35 kU_A_/L for at least one of the four aeroallergens analyzed using ImmunoCap™ (Thermo Fisher Scientific).

^e^Classified as sensitized if IgE level was ≥0.35 kU_A_/L for at least one of the 9 aeroallergens analyzed using Phadiatop™ followed by separate ImmunoCAP™ tests (Thermo Fisher Scientific).

**Table 2:** Primers used for quantitative assessment of H3 or H4 histone acetylation by PCR following chromatin immunoprecipitation (ChIP).

Gene	Forward primer	Reverse primer
*CD14*	ATCAGGGTTCACAGAGGA	GACCCCAAGACCCTACAC

*FOXP3*	ATCGTGAGGATGGATGCATTAATA	CCACTGGGAAGGTCCCTAGC

*HDAC4*	CTCAACACAAGCCTCCCAAG	GTGAGGGTGTGGGGTGTAG

*IFNG*	AATCCCACCAGAATGGCACAGGTG	GAACAATGTGCTGCACCTCCTCTGG

*IL13*	TGTGGGAGATGCCGTGGG	TCTGACTCCCAGAAGTCTGC

*RPL32*	GGAAGTGCTTGCCTTTTTCC	GGATTGCCACGGATTAACAC

*SH2B3*	TTGAGTGGGTGGGGCTAAAG	CCTACCAATCCCGCTAGTCC

**Table:** (a) Histopathology of the 171 placentas available from the participating 173 mothers

Lifestyle	Anthroposophic N = 24	Partly anthroposophic N = 105	Anthroposophic and partly anthroposophic N = 129	Non-anthroposophic N = 42	p^*∗*^
*Placenta histopathology*, n/N (%)					

Chorioamnionitis	9/24 (37.5%)	44/104 (42.3%)	53/128 (42.4%)	20/42 (47.6%)	0.59
Vasculitis chorion plate	1/24 (4.2%)	15/104 (14.4%)	16/128 (12.5%)	7/42 (16.7%)	0.60
Vasculitis umbilical cord	2/24 (8.3%)	9/103 (8.6%)	11/127 (8.5%)	2/42 (4.8%)	0.73
Funisitis	1/24 (4.2%)	3/103 (2.9%)	4/127 (3.1%)	1/42 (2.4%)	1.00
Villitis	4/24 (16.7%)	20/105 (19%)	24/129 (18.6%)	4/42 (9.5%)	0.23

*∗*p for comparisons of the combined anthroposophic and partly anthroposophic versus the nonanthroposophic lifestyle group by Fisher's exact test.

**Table:** (b) Sensitization during childhood in relation to placenta histopathology among families with any data both from placenta and child blood sample available (N=155).

*Children sensitized to food allergens and/or aeroallergens*, n/N	At 6 months	At 12 months	At 2 years	At 5 years
	12/116	19/121	21/126	34/110

*Placenta histopathology* n/N (p^*∗*^)				

Chorioamnionitis	7/12 (0.37)	10/19 (0.45)	9/21 (0.82)	13/34 (0.54)
Vasculitis chorion plate	2/12 (0.64)	4/19 (0.47)	3/21 (0.73)	7/34 (0.23)
Vasculitis umbilical cord	1/12 (0.60)	2/19 (0.73)	2/21 (0.49)	3/33 (0.60)
Funisitis	0/12 (1.0)	0/19 (1.0)	0/21 (1.0)	1/33 (0.79)
Villitis	2/12 (1.0)	2/19 (0.74)	5/21 (0.31)	5/34 (1.0)

*∗*Fisher's exact test.

## Data Availability

Demographic and histone acetylation data used to support the findings of this study are available as Supplementary data table and the histopathologic data are available from the corresponding author upon request.

## Supplementary Materials

Supplementary Materials
* Supplementary Table 1 A*. Histopathology according to sex of the child.* Supplementary Table 1 B*. Sensitization during childhood according to sex of the child in relation to placenta histopathology.* Supplementary Table 2*. Histone acetylation levels in 173 placentas in the different lifestyle groups, and in girls and boys within each group.* Supplementary data table*. Demographic and histone acetylation data (database).
