# Supplementary material for: Epigenetic Modifications in Placenta are Associated with the Child's Sensitization to Allergens
Source: Biomed Res Int. 2019 Apr 17;2019:1315257. doi: 10.1155/2019/1315257 (PMC6500694; doi:10.1155/2019/1315257)
Supplement: Supplementary Materials — Supplementary Table 1 A. Histopathology according to sex of the child. Supplementary Table 1 B. Sensitization during childhood according to sex of the child in relation to placenta histopathology. Supplementary Table 2. Histone acetylation levels in 173 placentas in the different lifestyle groups, and in girls and boys within each group. Supplementary data table. Demographic and histone acetylation data (database). [file 1315257.f1.zip › biomed research international - supplementary tables 1 & 2_2019-03-13_BMRI_2710577.docx]

**Supplementary table 1A.** **Histopathology according to sex of the child from the 171 placentas available from the participating 173 mothers.**

|  | **Girls**  **N = 92** | **Boys**  **N = 79** | **p*** |
| --- | --- | --- | --- |
| **Placenta histopathology, n/N (%)** | | | |
| Chorioamnionitis | 41/92 (44.6%) | 32/78 (41.0%) | 0.76 |
| Vasculitis chorion plate | 11/92 (12.0%) | 12/78 (15.4%) | 0.65 |
| Vasculitis umbilical cord | 7/90 (7.8%) | 6/79 (7.6%) | 0.69 |
| Funisitis | 3/90 (3.3%) | 2/79 (2.5%) | 0.69 |
| Villitis | 12/92 (13.0%) | 16/79 (20.3%) | 0.22 |

*Fisher´s exact test

| **Supplementary table 1B.** **Sensitization during childhood according to sex of the child in relation to placenta histopathology among families with any data available both from the placenta and child blood samples (N=155).** | | | | | | | | |
| --- | --- | --- | --- | --- | --- | --- | --- | --- |
| **Child sensitized to**  **food allergens and/or aeroallergens at (n/N)*** | **6 months**  **12/116**  **Girls Boys**** | | **12 months**  **19/121**  **Girls Boys**** | | **2 years**  **21/126**  **Girls Boys**** | | **5 years**  **34/110**  **Girls Boys**** | |
| **Placenta histopathology**, **n/N** | | | | | | | | |
| Chorioamnionitis | 3/6 | 4/6 | 4/9 | 6/10 | 2/7 | 7/14 | 7/18 | 6/16 |
| Vasculitis chorion plate | 1/6 | 1/6 | 1/9 | 3/10 | 0/7 | 3/14 | 4/18 | 3/16 |
| Vasculitis umbilical cord | 0/6 | 1/6 | 0/9 | 2/10 | 0/7 | 2/14 | 1/17 | 2/16 |
| Funisitis | 0/6 | 0/6 | 0/9 | 0/10 | 0/7 | 0/14 | 1/17 | 0/16 |
| Villitis | 0/6 | 2/6 | 0/9 | 2/10 | 1/7 | 4/14 | 2/18 | 3/16 |

*See Materials and Methods

**Fisher´s exact test: no significant values below 0.05.

**Supplementary table 2. Histone acetylation levels in 173 placentas in the different lifestyle groups, and in girls and boys within each group.**

|  | **Anthro**  **N=25** | **Partly anthro N=105** | **Anthro + partly N=130** | **Non-anthro**  **N=43** | **p*** |
| --- | --- | --- | --- | --- | --- |
| H3ac-*CD14* | 0.261 (0.012-0.988) | 0.471 (0.02-1.667) | 0.381 (0.014-1.646) | 0.501 (0.055-1.757) | 0.67 |
| H4ac-*CD14* | 0.528 (0.078-0.871) | 0.374 (0.123-0.775) | 0.38 (0.112-0.846) | 0.386 (0.165-0.499) | 0.38 |
| H3ac-*FOXP3* | 0.092 (0-0.309) | 0.258 (0-0.572) | 0.215 (0-0.52) | 0.131 (0-0.322) | 0.31 |
| H4ac-*FOXP3* | 0.037 (0-0.209) | 0.052 (0.012-0.202) | 0.051 (0.01-0.204) | 0.027 (0.009-0.126) | 0.32 |
| H3ac-*HDAC4* | 0.266 (0.036-0.623) | 0.49 (0.016-1.007) | 0.444 (0.017-0.942) | 0.1 (0-0.908) | 0.09 |
| H4ac-*HDAC4* | 1.248 (0.346-2.055) | 0.992 (0.073-1.579) | 1.022 (0.106-1.648) | 1.033 (0.2-1.7) | 0.98 |
| H3ac-*IFNG* | 0.025 (0-0.309) | 0.098 (0-0.584) | 0.075 (0-0.567) | 0.039 (0-0.435) | 0.53 |
| H4ac-*IFNG* | 0.063 (0.004-0.327) | 0.027 (0-0.204) | 0.035 (0-0.232) | 0.009 (0-0.102) | 0.12 |
| H3ac-*IL13* | 0.113 (0-0.988) | 0.025 (0-0.768) | 0.044 (0-0.831) | 0.035 (0-0.774) | 0.92 |
| H4ac-*IL13* | 0.051 (0.007-0.393) | 0.079 (0.002-0.421) | 0.075 (0.003-0.414) | 0.127 (0.032-0.516) | 0.23 |
| H3ac-*SH2B3* | 0.058 (0-0.621) | 0.264 (0-1.125) | 0.157 (0-1.088) | 0.075 (0-1.406) | 0.89 |
| H4ac-*SH2B3* | 0.059 (0-0.813) | 0.105 (0.008-0.4) | 0.101 (0-0.517) | 0.159 (0-0.382) | 0.98 |
| **Girls** | **N=13** | **N=52** | **N=65** | **N=28** |  |
| H3ac-*CD14* | 0.239 (0-0.912) | 0.282 (0-1.113) | 0.278 (0-0.988) | 0.555 (0.14-1.727) | 0.12 |
| H4ac-*CD14* | 0.303 (0.075-0.777) | 0.305 (0.061-0.719) | 0.303 (0.062-0.743) | 0.384 (0.182-0.545) | 0.92 |
| H3ac-*FOXP3* | 0 (0-0.262) | 0.196 (0-0.514) | 0.092 (0-0.483) | 0.119 (0-0.27) | 0.96 |
| H4ac-*FOXP3* | 0.114 (0-0.533) | 0.035 (0.011-0.17) | 0.037 (0.009-0.194) | 0.024 (0.005-0.09) | 0.26 |
| H3ac-*HDAC4* | 0.204 (0.036-0.486) | 0.321 (0-0.909) | 0.319 (0-0.899) | 0.106 (0-0.981) | 0.60 |
| H4ac-*HDAC4* | 1.248 (0.355-2.08) | 1.065 (0.127-1.622) | 1.078 (0.341-1.649) | 0.917 (0.103-1.94) | 0.64 |
| H3ac-*IFNG* | 0.267 (0-1.14) | 0.072 (0-0.658) | 0.094 (0-0.658) | 0.045 (0-0.344) | 0.60 |
| H4ac-*IFNG* | 0.05 (0.004-0.533) | 0.016 (0-0.197) | 0.026 (0-0.204) | 0.011 (0-0.066) | 0.43 |
| H3ac-*IL13* | 0.113 (0-0.496) | 0.013 (0-0.789) | 0.015 (0-0.609) | 0.094 (0-0.717) | 0.74 |
| H4ac-*IL13* | 0.057 (0.008-0.329) | 0.05 (0.001-0.512) | 0.055 (0.001-0.503) | 0.104 (0.033-0.435) | 0.26 |
| H3ac-*SH2B3* | 0.259 (0-1.536) | 0.208 (0-0.958) | 0.259 (0-1.031) | 0.125 (0-1.471) | 0.85 |
| H4ac-*SH2B3* | 0.043 (0-0.517) | 0.076 (0.008-0.267) | 0.073 (0-0.406) | 0.149 (0.008-0.276) | 0.54 |
| **Boys** | **N=12** | **N=53** | **N=65** | **N=15** |  |
| H3ac-*CD14* | 0.335 (0.093-1.634) | 0.707 (0.104-1.891) | 0.628 (0.104-1.864) | 0.222 (0.027-1.756) | 0.36 |
| H4ac-*CD14* | 0.583 (0.259-0.928) | 0.512 (0.198-1.475) | 0.532 (0.198-1.284) | 0.41 (0.145-0.479) | 0.34 |
| H3ac-*FOXP3* | 0.146 (0.061-0.371) | 0.375 (0.018-0.921) | 0.334 (0.031-0.619) | 0.131 (0-0.465) | 0.30 |
| H4ac-*FOXP3* | 0.026 (0-0.164) | 0.068 (0.014-0.228) | 0.059 (0.011-0.214) | 0.056 (0.012-0.206) | 0.99 |
| H3ac-*HDAC4* | 0.304 (0.052-0.676) | 0.535 (0.125-1.335) | 0.49 (0.099-1.098) | 0.028 (0-0.616) | 0.10 |
| H4ac-*HDAC4* | 1.131 (0.298-1.865) | 0.845 (0.055-1.387) | 0.886 (0.073-1.596) | 1.31 (0.328-1.536) | 0.60 |
| H3ac-*IFNG* | 0.011 (0-0.045) | 0.102 (0-0.426) | 0.067 (0-0.374) | 0 (0-0.557) | 0.71 |
| H4ac-*IFNG* | 0.115 (0.019-0.279) | 0.035 (0.003-0.228) | 0.047 (0.003-0.263) | 0.007 (0-0.146) | 0.27 |
| H3ac-*IL13* | 0.173 (0-1.23) | 0.121 (0-0.716) | 0.121 (0-0.952) | 0.009 (0-1.749) | 0.98 |
| H4ac-*IL13* | 0.043 (0.005-0.409) | 0.108 (0.01-0.338) | 0.083 (0.007-0.393) | 0.15 (0.034-0.522) | 0.50 |
| H3ac-*SH2B3* | 0.029 (0-0.361) | 0.351 (0-1.278) | 0.13 (0-1.107) | 0.041 (0-1.326) | 0.87 |
| H4ac-*SH2B3* | 0.288 (0-0.838) | 0.165 (0.008-0.508) | 0.165 (0.004-0.654) | 0.164 (0-0.5) | 0.68 |

Data are given as median (interquartile range).

*p for comparisons of combined anthro (anthroposophic) and partly anthro (partly anthroposophic) *vs* non-anthro (non-anthroposophic) lifestyle group. Mann-Whitney-Wilcoxon rank-sum test.

H3ac denotes histone H3 pan-acetylation; H4ac, histone H4 pan-acetylation.
